# Prehospital therapeutic hypothermia after cardiac arrest - from current concepts to a future standard

**DOI:** 10.1186/1757-7241-17-53

**Published:** 2009-10-12

**Authors:** Antti Kämäräinen, Sanna Hoppu, Tom Silfvast, Ilkka Virkkunen

**Affiliations:** 1 Critical Care Medicine Research Group, Department of Intensive Care Medicine, Tampere University Hospital, Tampere, Finland; 2 Department of Anaesthesia and Intensive Care, Helsinki University Hospital, Helsinki, Finland; 3 Faculty of Medicine, University of Tampere, Tampere, Finland; 4 Department of Surgery and Anaesthesia, Tampere University Hospital, Tampere, Finland

## Abstract

Therapeutic hypothermia has been shown to improve survival and neurological outcome after prehospital cardiac arrest. Existing experimental and clinical evidence supports the notion that delayed cooling results in lesser benefit compared to early induction of mild hypothermia soon after return of spontaneous circulation. Therefore a practical approach would be to initiate cooling already in the prehospital setting.

The purpose of this review was to evaluate current clinical studies on prehospital induction of mild hypothermia after cardiac arrest. Most reported studies present data on cooling rates, safety and feasibility of different methods, but are inconclusive as regarding to outcome effects.

## Background

Following successful resuscitation from cardiac arrest, induced mild therapeutic hypothermia (TH) at 32 to 34°C for 12 to 24 hours has been shown to improve overall survival and neurological outcome[1,2]. These results are derived from prehospital cardiac arrest victims resuscitated from ventricular fibrillation (VF), and current resuscitation guidelines of the International Liaison Committee on Resuscitation (ILCOR) promote induction of TH in this patient subgroup[3]. However, more recent evidence has now shown that the treatment is beneficial in cases with non-VF initial rhythm also[4]. Recently published Scandinavian guidelines recommend to consider TH in these cases as well if active treatment is chosen[5].

The potential mechanisms of mild hypothermia as a protecting and preserving factor after cardiopulmonary resuscitation have been summarized by the Task Force on Scandinavian Therapeutic Hypothermia Guidelines[5]. Most of the deleterious reactions suppressed by TH are either initiated at or exacerbated rapidly after return of spontaneous circulation (ROSC) following successful resuscitation. There is experimental evidence showing that a delay in cooling results in lesser benefit [6] and, following successful resuscitation, TH is recommended to be induced as soon as possible[3,5]. Following prehospital cardiac arrest, rapid induction of mild hypothermia is best achieved by emergency medical service (EMS) personnel prior to and during transfer to hospital. In this article, we review the current evidence on prehospital induction of mild hypothermia in the context of sudden cardiac arrest.

## Methods

The databases PubMed, MEDLINE, CINAHL and EMBASE were searched for original articles in English through August 2009 with the following search terms: (prehospital OR pre-hospital OR out-of-hospital OR out of hospital OR OOHCA) AND (cardiac arrest OR heart arrest OR resuscitation OR CPR OR cardiopulmonary resuscitation) AND (therapeutic hypothermia OR mild hypothermia OR induced hypothermia) and limited to adult (age 19+ years) human studies. Titles and abstracts of studies investigating the use of induced hypothermia in the prehospital setting in association with cardiac arrest were hand-searched for potential relevance. The reference lists of these articles were further screened for potentially relevant articles. Articles on accidental or in-hospital induced hypothermia were excluded.

## Review

**Figure 1:**
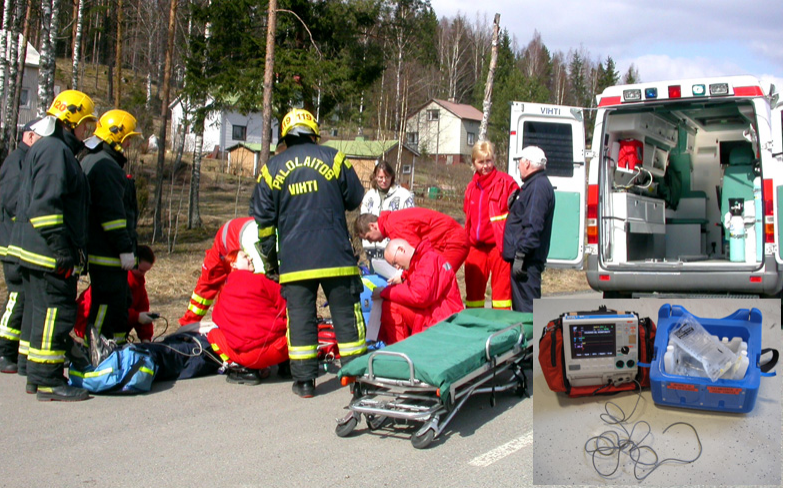
**All you need is this**. Prehospital induction of therapeutic hypothermia with infusion of ice-cold fluid. Small picture: a biphasic defibrillator/monitor with a temperature probe and ice cold fluids in a medical refrigeration box.

The first report on prehospital cooling is by Callaway et al in 2002[7]. In their study, ice was applied already during cardiopulmonary resuscitation (CPR) to the heads and necks of 9 patients with a control group of 13 patients. No difference in the rate of cooling was observed between the groups and the method was not found feasible. In 2004 our group reported a feasibility trial using post ROSC infusion of large volume ice cold fluid (LVICF, Figure 1)[8]. In that trial, 30 ml/kg of +4°C Ringer's solution was infused after ROSC at a rate of 100 ml/min with a target temperature of 33°C. In a cohort of thirteen patients, a significant decrease in oesophageal temperature was observed, with a mean decrease of 1.9°C compared to the temperature prior to the onset of infusion. A transient episode of hypotension was observed in one patient, but otherwise the treatment was well tolerated.

The first randomized controlled trial (RCT) of prehospital cooling using LVICF was reported by Kim et al in 2007[9]. Adult victims of non-traumatic cardiac arrest regardless of the initial rhythm were included, resulting in 125 patients randomized either to field cooling or conventional treatment. In the treatment group, a fixed volume of 2 litres of cold (+4°C) saline was intended to be administrated, but only 12 patients received the target volume. Despite this, among survivors to hospital admission, a significant oesophageal temperature decrease of 1.24°C (SD ± 1.09, n = 54) was observed in the treatment group compared to a 0.10°C (SD ± 0.94, n = 36) increase in the control group (p < 0.0001). The authors report no increase in the number of adverse events associated with field cooling.

We reported similar results in our subsequent RCT on prehospital cooling[10]. Of 44 patients screened, 19 were cooled using LVICF and 18 patients received conventional fluid therapy. Layperson CPR was more common in the treatment group, but otherwise the groups were comparable regarding baseline characteristics. The mean (± SD) infused volume of cold fluid per patient in the treatment group was 2370 (± 500) ml, which resulted in a mean decrease in nasopharyngeal temperature of 1.5 (± 0.8)°C. At the time of hospital admission, the mean (± SD) nasopharyngeal temperature was markedly lower in the hypothermia group compared to the control group; 34.1 ± 0.9°C vs. 35.2 ± 0.8°C, respectively (p < 0.001). Otherwise, there were no significant differences between the groups regarding safety such as the rate of rearrest, haemodynamic stability or pulmonary oedema. The study was not designed nor powered to investigate secondary outcome measures such as neurological outcome or mortality[10].

A French study retrospectively compared 22 patients cooled using LVICF in the prehospital setting to 77 conventionally treated patients[11]. In this non-randomized trial the aim was to evaluate the feasibility of an immediate prehospital cooling protocol following ROSC. Cooling using LVICF was found to be a feasible and safe method with a mean cooling rate of -1.7 C/h and no significant increase in the rate of adverse effects in the cooling group. Long-term survival and neurological outcome one year after cardiac arrest were reported. The outcome was better in the control group, but the difference was not statistically significant due to the small size of hypothermia group.

The feasibility of prehospital cooling using self-adhesive cooling pads was studied by Uray et al[12]. Cooling was initiated after ROSC and continued in hospital with a target temperature of 33 to 34°C for 24 hours. 15 patients were included and 14 underwent the whole protocol. The overall median rate of cooling was 3.3 (IQR 2.0-4.0)°C/h, resulting in reaching the target temperature in hospital approximately 91 minutes after ROSC. Although the absolute temperature decrease at the time of hospital admission is not presented, it is evident from a graphical presentation in this study that rapid cooling to target temperature was not achieved in the prehospital setting. On the other hand, the treatment was found feasible and no adverse events associated with the cooling process were observed. A further benefit of this method of cooling was that it was seamlessly continued from the prehospital setting to the ICU.

Another application of external cooling is the use of a cranial cooling cap. The out-of-hospital feasibility of this approach was studied by Storm et al[13]. In the final analysis, elective cranial cooling was initiated after ROSC in 20 patients compared to 25 patients serving as a non-randomized control group. A mild decrease (-1.1°C) in tympanic temperature was observed in the treatment group, which was statistically significant compared to the control group (p < 0.001).

The main characteristics and results of the presented studies are outlined in Table 1.

**Table 1:** Summary of clinical trials on prehospital cooling.

	**Method**	**EMS setting**	**Number of patients (hypothermia)**	**Control group**	**Intra-arrest cooling**	**Mean ΔT in hypothermia group at hospital admission**	**ΔT Difference to control group**	**Temperature measurement**	**Adverse events**
Virkkunen et al 2004 [8]	LVICF	Physician staffed	13	No	No	-1.9 (Range -3.1 to +0.4°C)	NA	Oesophageal	1 transient hypotension
Kim et al 2007 [9]	LVICF	Paramedic	63	62	No	-1.24° SD ± 1.09	p < 0.0001	Oesophageal	NS
Kämäräinen et al 2009 [10]	LVICF	Physician	19	18	No	-1.5 (± 0.8)°C	p < 0.001	NP	NS
Hammer et al 2009 [11]	LVICF	Physician	22	77	No	Median: -1.3°C	p = 0.06	Rectal	NS
Uray et al 2008 [12]	Cooling pads	Physician	15	No	No	Median cooling rate: 3.3 (2.0-4.0)°C/h ^†^	NA	Oesophageal	No
Storm et al 2008 [13]	Cooling cap	Physician	20	25	No	Median -1.1°C	p < 0.001	Tympanic	No
Callaway et al 2002 [7]	External cranial cooling	Physician staffed	9	13	Yes	-0.07°(SD ± 0.06)°C/min*	NS	NP, Oesophageal	No
Bruel et al 2008 [15]	LVICF	Physician	33	No	Yes	2.1 (SD ± 0.29)°C	NA	Oesophageal	1 pulmonary oedema
Kämäräinen et al 2008 [16]	LVICF	Paramedic	17	No	Yes	-1.34 (Range 0 to -2.7°C)	NA	NP	5 cases of rearrest

EMS; emergency medical service, * Temporal rate of cooling presented only, LVICF; large volume ice cold fluid, ^† ^Cooling rate presented only. NS; not significant, NP; nasopharyngeal, NA; not applicable.

In 2008, several reports on prehospital induction of mild hypothermia were published. Our small pilot study [14] on intra-arrest and post ROSC cooling using LVICF was followed by a similar and larger study by Bruel et al [15] and our final results [16]. In the study by Bruel et al, 33 patients were included and 20 of these regained spontaneous circulation. A mean oesophageal temperature decrease of 2.1 (SD ± 0.29)°C was observed. The mean rate of infusion was 67 ml/min and the volume of cold saline per patient was 2 litres[15]. Pulmonary oedema was observed in one patient and the infusion of cold saline was interrupted after 1500 ml. No cases of rearrest or arrhythmia were observed. Cooling was continued in hospital and 4 patients out of 11 surviving to intensive care unit (ICU) admission were alive after 6 months, three with a CPC [17] score ≤ 2.

In our material of 17 patients paramedics initiated cooling using infusion of cold fluid during CPR and after ROSC at an overall calculated rate of 57 ± 21 ml/min (95% CI) with a target temperature of 33°C. The mean infused volume of cold fluid per patient was 1571 ± 517 ml and resulted in a mean admission temperature of 33.83 ± 0.77°C (n = 11, -1.34°C decrease compared to initial nasopharyngeal temperature)[16]. No apparent increase in the rate of rearrest or haemodynamic instability was observed, and the treatment was easily carried out by paramedics.

## Discussion

As is evident from above, the current studies on prehospital induction of TH reporting the use of either external cooling or infusion of cold fluid have mainly focused on the cooling effects and feasibility. Two of these studies are randomized controlled trials [9,10], but they are insufficient in power to imply any significant outcome benefit effect associated with prehospital cooling. A major limitation in most of these studies is that TH is not systematically continued in the post resuscitation care occurring in hospital. Therefore it is not possible to evaluate the benefits of prehospital cooling alone as the effect of TH has been shown to necessitate a cooling period of at least 12 to 24 hours[1,2]. In the future, a properly controlled study setting would also need to take into account relevant patient characteristics (e.g. initial cardiac rhythm), delays, quality of resuscitation and post resuscitation treatment, but even with this approach a proper blinded treatment might prove cumbersome.

A pulmonary artery catheter is generally accepted as the golden standard for core temperature measurement. However, in a recent review article both oesophageal and nasopharyngeal temperature measurement were addressed as highly accurate and fast methods to monitor core temperature during therapeutic hypothermia[18]. Oesophageal temperature measurement probably reflects core temperature most reliably, although it is subject to misplacement and the proximity of large vessels might be a source of bias at least when infusions of LVICF are used. Nasopharyngeal temperature probes are feasible but also prone to misplacement. Tympanic temperature is easy to measure, but does not necessarily correlate to core or cerebral temperature and is potentially affected by focal cooling such as a cooling cap [16-20].

In the present studies a significant change in core temperatures has been observed, be it a difference between the initial and admission temperature or difference between groups. Whether the statistically significant drop in temperature also represents a clinical significant improvement is still unknown. It would be easy to repeat the often heard mantra of "further studies are needed, a sufficiently powered randomized controlled trial is necessitated" but is this really so? Schefold [21] and colleagues have already questioned the necessity of a large RCT to justify prehospital cooling as this might be considered unethical in the control group due to already observed benefits of cooling in general. Still, what can be said is that current evidence regarding this treatment is insufficient to either strongly support or refute it. An optimistic rationalisation on the mechanisms of cerebral ischaemia and protective hypothermia derived from both clinical and experimental studies would support early cooling already during cardiac arrest, let alone after ROSC[5,15,16,22-24].

A survey on the implementation rate of prehospital cooling in the United States proposed that the lack of specific guidelines was not the main reason for not providing prehospital cooling[25]. One of the main reasons was the lack of ideal equipment to initiate cooling. This emphasizes the need for a simple method of cooling feasible in the prehospital setting. Infusion of LVICF and external cooling may both be effective and non-invasive, but which is superior? The answer might, in fact, be a combination of both. LVICF provides effective core cooling, but to which extent this is mediated to the cerebrum is unknown. The cooling effect of intravenous cold fluid to the cortical tissue is somewhat dependent on adequate cerebral perfusion, which is known to be deranged in the early post resuscitation phase[26]. Selective external cranial cooling might add to the effect of LVICF via conductive cooling and thus provide enhanced protection of the cortical cerebral tissue. On the other hand, external conductive cooling might not initially provide sufficient protection of the particularly vulnerable deep regions of the brain [27], to which infusion of LVICF might be capable.

In a very recent retrospective study, the effect on LVICF on respiratory function was studied. The authors conclude that infusion of LVICF does not cause further deterioration in respiratory function after cardiac arrest[28]. Also, an experimental study on cold fluids demonstrated that cold infusion fluids begin to warm toward ambient temperature, but the rate is not rapid and thus unlikely to be of clinical significance[29].

Finally, protocol descriptions and feasibility reports mainly utilising the infusion on LVICF have been published, however, with no additional evidence to promote prehospital cooling in terms of improved outcome [30-32]. Thus it is understandable that given the occasionally limited resources of prehospital resuscitation and staff, some authorities recommend basic resuscitation skills and manoeuvres such as effective chest compressions and rapid defibrillation proven to be beneficial to be prioritized over cooling[33]. On the other hand, after initial successful resuscitation, induction of mild hypothermia in the prehospital phase might urge this treatment to be continued in the hospital also. This might increase the implementation of the treatment in general, although one study addressing this aspect does not support the notion [9].

## Conclusion

In conclusion, a handful of studies on prehospital cooling have been published, most reporting an effective decrease in temperature regardless of the cooling method. None of the reports describe significantly increased rates of adverse events, such as rearrest, haemodynamic instability or bleeding. The published studies are either underpowered or due to study design do not allow conclusions regarding effects on outcome to be drawn, but the feasibility of early cooling is well documented. In the light of current evidence, it does seem safe to initiate cooling already in the prehospital phase, and the rationale regarding the protective mechanisms of early cooling supports this. We consider it justifiable to implement prehospital cooling even in the absence of unambiguous evidence to support this practice, rather than leave the patients without a potentially beneficial treatment during the wait for such evidence.

## Competing interests

The authors declare that they have no competing interests.

## Authors' contributions

AK, TS and IV designed the study, AK and IV performed the literature search, AK, SH and IV reviewed the articles. All authors drafted and revised the manuscript, as well as approved the final version.
